# Toward a Behavioral Reserve Model in Amyotrophic Lateral Sclerosis

**DOI:** 10.1002/ana.78315

**Published:** 2026-07-30

**Authors:** Francesca Palumbo, Barbara Iazzolino, Cristina Moglia, Umberto Manera, Enrico Matteoni, Sara Cabras, Giorgio Pellegrino, Emilio Minerva, Vita Passidomo, Rosario Vasta, Gabriele Mora, Fabrizio D’Ovidio, Antonio Canosa, Andrea Calvo, Adriano Chiò

**Affiliations:** ^1^ ALS Center, “Rita Levi Montalcini” Department of Neuroscience, University of Torino Turin Italy; ^2^ Department of Neurology 1, Azienda Ospedaliero – Universitaria Città della Salute e della Scienza of Torino Turin Italy; ^3^ Institute of Cognitive Sciences and Technologies, National Research Council Rome Italy

## Abstract

**Objective:**

Behavioral impairment is common in amyotrophic lateral sclerosis (ALS) and strongly affects autonomy, caregiver burden, and outcomes, yet predictors of vulnerability remain unclear. We investigated whether premorbid regulatory traits and socio‐educational exposures are associated with behavioral phenotypes in ALS within a behavioral reserve framework.

**Methods:**

We analyzed 965 consecutively assessed patients with ALS from a prospective tertiary‐center cohort. Behavioral impairment was measured using the Frontal Systems Behavior Scale (FrSBe; family‐rated before and after onset) and, in a subset (n = 633), the Edinburgh Cognitive and Behavioural ALS Screen–Carer Interview (ECAS‐CI). Theory‐driven hierarchical logistic regression models tested associations between behavioral outcomes and premorbid behavioral regulation, education, occupation, and a Social Interaction Index, including interaction effects. Prespecified sensitivity analyses addressed potential bias in premorbid estimates.

**Results:**

Premorbid behavioral regulation showed the strongest associations with behavioral impairment across all FrSBe models. Higher combined educational attainment and social exposure were associated with lower odds of impairment, with a significant interaction across multiple behavioral domains. These patterns persisted in restricted sensitivity analyses. Reserve proxies were not significantly associated with ECAS‐CI total impairment, although domain‐specific effects were observed.

**Interpretation:**

These findings provide empirical support for a behavioral reserve framework in ALS, in which premorbid regulatory traits and socio‐educational exposures are associated with behavioral vulnerability. Although causal inference is limited, reserve‐related factors may contribute to non‐motor heterogeneity in ALS and may inform future approaches to early behavioral risk stratification. ANN NEUROL 2026;100:934–947

Cognitive reserve (CR) refers to the capacity to maintain function despite neuropathology^1^, ^2^ and is well established in neurodegenerative disorders including Alzheimer's disease (AD) and frontotemporal dementia (FTD). Higher educational attainment and occupational complexity are associated with delayed clinical expression and greater neurodegenerative burden at equivalent clinical severity, reflecting compensatory mechanisms within distributed neural networks.^3^, ^4^, ^5^ A systematic review confirmed partial support for CR in FTD, with education as the most consistent proxy and evidence of increased hypometabolism at comparable clinical stages.^6^ In amyotrophic lateral sclerosis (ALS), CR has been shown to modulate cognitive performance and disease trajectory^7^, ^8^, ^9^ but appears to have limited or indirect effects on behavioral symptoms.^7^, ^9^

This limitation is critical because ALS frequently involves frontolimbic networks. Up to 50% of patients exhibit cognitive and/or behavioral changes, and approximately 15% develop an overt frontotemporal syndrome, including apathy, disinhibition, loss of empathy, and perseveration. These disturbances arise from degeneration of neural systems governing social behavior and emotional regulation, primarily involving salience and medial frontal networks.^10^, ^11^, ^12^ These systems are partly distinct from the frontoparietal circuits typically implicated in CR, suggesting that a reserve construct limited to cognition is insufficient to account for behavioral heterogeneity in ALS.

We hypothesize that behavioral reserve (BvR) may represent a distinct dimension of resilience, defined as the capacity to maintain adaptive social and emotional functioning despite neurodegeneration affecting frontolimbic systems. Evidence from FTD supports this concept: education and occupation alone incompletely capture reserve processes, and occupational profiles emphasizing social interaction and cognitive control modulate neural vulnerability.^13^ Neuroimaging studies are consistent with the possibility of reserve mechanisms relevant to behavioral phenotypes,^14^, ^15^ and the original BvR hypothesis already highlighted the need to move beyond purely cognitive proxies.^5^ More broadly, reserve appears to depend on multidimensional and interacting factors, including socioeconomic context^16^ and lifestyle engagement.^17^

A key unresolved issue is measurement. Proxy‐based approaches provide only indirect estimates of reserve, whereas residual approaches, which operationalize reserve as the variance in behavioral outcomes not explained by neurodegeneration, risk circularity, or bias when the predictors used to define the residual are themselves correlated with the outcome through pathways unrelated to reserve (i.e. when education or other proxies independently influence behavioral outcomes beyond reserve mechanisms, the residual no longer cleanly isolates the reserve component).^18^ These limitations are particularly relevant in behavioral domains, where premorbid personality and regulatory capacity are likely central determinants. Instruments capturing change from baseline offer a direct means of modeling this trait component.

In this study, we test a multidimensional BvR framework in a large, prospectively characterized ALS cohort. BvR comprises 2 main dimensions: socio‐educational exposure, indexed by (1) general reserve proxies (education and occupation); (2) socio‐emotional exposure (occupational social interaction and relationship status); and premorbid behavioral regulatory capacity.

## Methods

### Participants

All patients with ALS who underwent cognitive and behavioral screening at the Turin ALS Expert Centre from January 2009 onward were eligible. Testing is generally performed within 2 months of diagnosis. Patients were diagnosed according to the revised El Escorial or Gold Coast criteria.^19^, ^20^ Our complete neuropsychological battery encompasses executive function, verbal and visual memory, attention and working memory, visuospatial function, language, social cognition, and behavior^21^ (for a complete list of tests see Supplementary Methods).

Patients carrying the *C9orf72* pathological expansion were excluded from analyses. For this study, 965 patients with complete Frontal Systems Behavior Scale (FrSBe) assessments were included in the regression models. Among the 965 patients, 633 also completed the Edinburgh Cognitive and Behavioural ALS Screen–Carer Interview (ECAS‐CI), which was introduced into the battery from 2016 onward. The 2 cohorts overlap: all ECAS‐CI patients also completed the FrSBe.

### Behavioral Assessment

The FrSBe^22^, ^23^, ^24^, ^25^, ^26^ is a 46‐item informant‐rated scale quantifying behaviors linked to frontal‐subcortical circuit dysfunction across 3 subscales: apathy, disinhibition, and executive dysfunction. Both the “before” (premorbid) and “after” (current) Family Forms were completed by a close caregiver, providing an informant‐based estimate of behavioral change from baseline. For this study, we considered the change in points for the total score and each of the 3 domains of FrSBe (apathy, disinhibition, and executive) from the before disease to the disease scores. A score ≥ 65 on the total or any subscale indicates clinically significant impairment. If a subject had scores reflecting a frontal systems abnormality both in the premorbid and in the post‐illness forms, he/she was considered pathological only if there was an increase of ≥10 points between the 2 forms.

The ECAS‐CI^27^ is an informant‐based interview assessing threshold‐level personality and social conduct changes across 5 domains: disinhibition, apathy or inertia, loss of sympathy/empathy, perseverative/compulsive behavior, and hyperorality/dietary changes. The Italian version has been validated.^28^, ^29^ Critically, the ECAS‐CI does not include a premorbid assessment, measuring absolute symptom presence rather than change from baseline. In addition to the 5 domains, the ALS Psychosis Screen checks for the presence of delusions and/or hallucinations. Because only 25 patients in our cohort were positive in this subscale and most of them carried the *C9ORF72* expansion, this subscale was not included in the analyses.

### Operationalization of Behavioral Reserve

BvR was operationalized as a multidimensional construct with 4 components. First, general CR proxies included years of formal education (a widely accepted proxy for intellectual enrichment) and occupation level. The latter was classified on a 5‐point scale^4^ (see Supplementary Methods), ranging from unskilled labor to senior management/academic positions. Second, socio‐emotional regulatory capacity was indexed by the Social Interaction Index (SII),^30^ which maps International Standard Classification of Occupations (ISCO)‐08 codes to relational complexity. This measure quantifies the interpersonal intensity of a job, thereby capturing the “social training” an individual has accumulated. Conjugal status (partnered vs single/unpartnered) was also included, as it reflects sustained, ecologically valid social engagement. Third, the trait/regulatory component comprised the premorbid FrSBe score (total and subscale‐specific), providing an estimate of baseline behavioral regulation prior to ALS onset. In addition, a history of affective disorder (chronic depression or bipolar disorder) was included as an index of premorbid vulnerability in emotional regulation.

The premorbid and post‐disease FrSBe scores are conceptually and temporally distinct. The “before” form captures the informant's recall of the patient's habitual personality and behavioral regulation prior to symptom onset, whereas the “after” form reflects current clinical status. The premorbid score was treated analytically as an independent predictor, although shared informant‐method variance cannot be fully excluded. This approach is consistent with the broader reserve literature, in which premorbid proxies such as education and occupation are accepted predictors of cognitive outcomes despite partial covariance and overlapping variance, provided the theoretical rationale is explicit and domain‐specific.^6^, ^18^

### Statistical Analysis

Theory‐driven hierarchical binary logistic regression with forced entry was used to predict clinical impairment separately for the FrSBe total score and each subscale, and for the ECAS‐CI total and each of its 5 subscales, yielding 10 models in total. Predictors were entered in 5 prespecified blocks: (1) demographic covariates (age and sex); (2) clinical covariates (disease duration in months and ALS Functional Rating Scale‐Revised [ALSFRS‐R] total score at time of testing; site of onset [bulbar/spinal]), entered to control for motor severity, motor phenotype, and time from onset before testing; (3) general reserve proxies (education and occupation level); (4) socio‐emotional exposure (SII, marital status, and Education_c × SII_c interaction); and (5) trait/regulatory component (premorbid FrSBe subscale score and depression history); no stepwise selection was applied. Prior to analysis, years of education and the SII were mean‐centered by subtracting each variable's sample mean, yielding Education_c and SII_c. In accordance with prespecified analytic priorities, the FrSBe total score model was designated as the primary confirmatory outcome, reflecting its status as the broadest and most established measure of frontal behavioral impairment in this cohort. The 3 FrSBe subscale models (apathy, disinhibition, and executive dysfunction) were designated secondary confirmatory analyses, testing domain‐specific hypotheses within the same instrument. The 6 ECAS‐CI models (total score and 5 subscales) were designated exploratory, given the smaller sample and shorter observation window (see Behavioral Assessment for instrument‐level rationale); findings from these models are presented as hypothesis‐generating and interpreted accordingly. All models entered the same candidate predictor set: age, sex, site of onset (0 = spinal and 1 = bulbar), marital status (0 = partnered and 1 = single/unpartnered), depression history (0 = no history of depression or bipolar disorder and 1 = history of depression or bipolar disorder), SII_c, Education_c, the Education_c × SII_c interaction, and occupation level. FrSBe models additionally included the premorbid FrSBe subscale‐specific score. Results are reported as odds ratios (ORs) with 95% confidence intervals; significance was set at *p* < 0.05 for confirmatory models. No post hoc correction for multiple comparisons was applied across the 10 models. The prespecified confirmatory/exploratory hierarchy (4 FrSBe models designated confirmatory and 6 ECAS‐CI models designated exploratory) provides partial protection against type I error inflation by concentrating inferential weight on the primary and secondary outcomes; findings from the exploratory ECAS‐CI models should therefore be interpreted with appropriate caution given the number of tests performed. All analyses were performed in SPSS version 30.

### Standard Protocol Approvals, Registrations, and Patient Consents

The study was approved by the Ethics Committee of the ALS Expert Center of Torino (Comitato Etico Azienda Ospedaliero‐Universitaria Città della Salute e della Scienza, Torino, Italy, #0036344, #0038876, and #0064510). Patients and controls provided written informed consent before enrollment. The database was anonymized according to Italian law for the protection of privacy.

## Results

### Sample Characteristics and Prevalence of Behavioral Involvement

Of 965 patients, 310 (32.2%) had a pathological total score. Among the 633 patients assessed with ECAS‐CI in the regression analyses, 286 (45.2%) showed impairment in the total behavioral score. The 2 cohorts were broadly comparable in terms of age (FrSBe: mean = 65.78 years, SD = 11.32 and ECAS‐CI: mean = 65.52 years, SD = 11.48), sex distribution (42.5% female patients in both), educational attainment (FrSBe: mean = 9.77 years, SD 4.32 and ECAS‐CI: mean = 10.05 years, SD = 4.29), occupation level (FrSBe: mean = 2.27, SD = 0.83 and ECAS‐CI: mean = 2.29, SD = 0.86), and SII (FrSBe: mean = 0.534, SD = 0.169 and ECAS‐CI: mean = 0.536, SD = 0.169). Most patients in both cohorts were partnered (FrSBe: 95.8% and ECAS‐CI: 96.4%), and a minority had a premorbid history of affective disorder (FrSBe: 5.3% and ECAS‐CI: 5.2%). Demographic and clinical characteristics are reported in Table 1.

**TABLE 1:** Demographic and Clinical Characteristics of the FrSBe and ECAS‐CI Cohorts

Variables	FrSBe Cohort			ECAS‐CI Cohort		
	Total (n = 965)	F (n = 410)	M (n = 555)	Total (n = 633)	F (n = 269)	M (n = 364)
Demographic characteristics						
Age, yr — mean (SD)	65.78 (11.32)	66.57 (10.92)	65.20 (11.58)	65.52 (11.48)	66.24 (10.85)	64.98 (11.92)
Sex, n (%)						
F	410 (42.5)	—	—	269 (42.5)	—	—
M	555 (57.5)	—	—	364 (57.5)	—	—
Marital status, n (%)						
Partnered	924 (95.8)	393 (95.9)	531 (95.7)	610 (96.4)	260 (96.7)	350 (96.2)
Single/unpartnered	41 (4.2)	17 (4.1)	24 (4.3)	23 (3.6)	9 (3.3)	14 (3.8)
History of affective disorder, n (%)	51 (5.3)	33 (8.0)	18 (3.2)	33 (5.2)	22 (8.2)	11 (3.0)
Reserve proxies						
Education, yr — mean (SD)	9.77 (4.32)	9.32 (4.40)	10.11 (4.24)	10.05 (4.29)	9.55 (4.33)	10.42 (4.23)
Occupation level — mean (SD)	2.27 (0.83)	2.16 (0.84)	2.36 (0.81)	2.29 (0.86)	2.14 (0.85)	2.39 (0.85)
SII – mean (SD)	0.534 (0.169)	0.510 (0.194)	0.553 (0.146)	0.536 (0.169)	0.520 (0.191)	0.547 (0.151)
Behavioral assessment						
FrSBe impairment (total score ≥ 65), n (%)	310 (32.2%)	129 (33.8%)	171 (30.8%)	—	—	—
ECAS‐CI total score impairment, n (%)	—	—	—	286 (45.2)	118 (43.9)	168 (46.2)

*Note*: Occupation level classified on a 5‐point scale (Borroni et al, 2009): 0 = no occupation; 1 = unskilled laborer/housewife; 2 = skilled laborer/tradesperson/lower‐level employee; 3 = mid‐level management/academic specialist; and 4 = senior management/senior academic. Social Interaction Index derived from ISCO‐08 occupational codes (Sostero et al, 2023); higher scores indicate greater relational complexity of the occupational role. History of affective disorder = premorbid chronic depression or bipolar disorder. FrSBe impairment is defined as total Family Form score ≥ 65. ECAS‐CI impairment defined as any subscale meeting clinical threshold. Marital status inverse‐coded in regression analyses (1 = single/unpartnered); history of affective disorder inverse‐coded (1 = no history). The FrSBe cohort (n = 973) includes all patients assessed from 2010 onward; the ECAS‐CI cohort (n = 633) includes patients assessed from 2016 onward. The 2 cohorts overlap: all ECAS‐CI patients also completed the FrSBe.

ALS = amyotrophic lateral sclerosis; ECAS‐CI = Edinburgh Cognitive and Behavioural ALS Screen Carer Interview; FrSBe = Frontal Systems Behavior Scale; ISCO‐08 = International Standard Classification of Occupations; SD = standard deviation; SSI = Social Interaction Index.

### FrSBe Models

Across all 4 FrSBe models, the premorbid domain‐specific FrSBe subscale score was the strongest and most consistent predictor of post‐disease impairment (all *p* < 0.001), with effect sizes increasing from the total score model (Exp(B) = 1.041; Table 2) through apathy (Exp(B) = 1.040; Table 3) and disinhibition (Exp(B) = 1.052; Table 4) to executive dysfunction (Exp(B) = 1.052; Table 5). Neither disease duration nor ALSFRS‐R total score reached significance in any of the 4 FrSBe models (disease duration: *p* range = 0.305–0.963 and ALSFRS‐R: *p* range = 0.075–0.373), suggesting that the observed associations were not fully explained by measured differences in motor severity or time from symptom onset. The bulbar site of onset resulted to be significant only in the apathy model (*p* = 0.035, Exp(B) = 1.458).

**TABLE 2:** Final Model: FrSBe Total Score – Binary Logistic Regression

Variables	*B*	SE	Wald	df	*p*‐value	Exp(B)	95% Confidence Interval Exp(B)	
							Lower	Upper
Sex, F = 1	−0.047	0.167	0.079	1	0.779	0.954	0.687	1.325
Age	0.026	0.009	9.595	1	0.002	1.027	1.010	1.044
ALSFRS‐R total score	−0.219	0.171	1.644	1	0.200	0.804	0.575	1.123
Disease duration, mo	0.000	0.007	0.002	1	0.963	1.000	0.986	1.014
Education_c, yr	−0.003	0.023	0.019	1	0.890	0.997	0.952	1.044
Occupation level	−0.147	0.126	1.355	1	0.244	0.864	0.675	1.105
SII_c	−0.247	0.541	0.209	1	0.647	0.781	0.270	2.254
Marital status, single = 1	−0.477	0.467	1.043	1	0.307	0.621	0.248	1.550
Education_c × SII‐c	−0.222	0.099	4.995	1	0.025	0.801	0.660	0.973
Depression, yes = 1	0.478	0.317	2.270	1	0.132	1.613	0.866	3.005
FrSBe premorbid total	0.040	0.006	38.867	1	< 0.001	1.041	1.028	1.055
Constant	−4.982	1.025	23.716	1	< 0.001	0.007		

*Note*: Theory‐driven hierarchical forced‐entry binary logistic regression (all predictors entered simultaneously). Bold red *p* values indicate *p* < 0.05. Depression coded 1 = history of affective disorder. Marital status coded 1 = single/unpartnered. Sex coded 1 = female. Education × SII = interaction between years of education and SII. Predictors entered in 5 prespecified blocks: (1) age, sex; (2) disease duration, ALSFRS‐R total score; (3) education, occupation level; (4) SII_c, marital status, Education_c × SII_c; and (5) premorbid FrSBe subscale score and depression history. All blocks used forced entry. Premorbid FrSBe total score = Family Form “before” total. FrSBe impairment criterion: total score ≥ 65 (for details see the text). ALS = amyotrophic lateral sclerosis; ALSFRS‐R = ALS Functional Rating Scale‐Revised; df = degree of freedom; Exp(B) = odds ratio; FrSBe = Frontal Systems Behavior Scale; SII = Social Interaction Index.

**TABLE 3:** Final Model: FrSBe Apathy Subscale – Binary Logistic Regression

Variables	*B*	SE	Wald	df	*p*‐value	Exp(B)	95% Confidence Interval Exp(B)	
							Lower	Upper
Sex, F = 1	0.107	0.151	0.496	1	0.481	1.113	0.827	1.497
Age	0.019	0.007	6.877	1	0.009	1.020	1.005	1.034
ALSFRS‐R total score	−0.279	0.156	3.179	1	0.075	0.757	0.557	1.028
Disease duration, mo	−0.001	0.007	0.032	1	0.859	−0.999	0.986	1.012
Site of onset ((bulbar = 1)	0.560	0.212	6.115	1	0.035	1.458	1.031	1.766
Education_c, yr	−0.017	0.022	0.658	1	0.417	0.983	0.942	1.025
Occupation level	−0.134	0.114	1.388	1	0.239	0.874	0.700	1.093
SII_c	−0.413	0.489	0.714	1	0.398	0.662	0.254	1.724
Marital status, single = 1	−0.119	0.380	0.098	1	0.755	0.888	0.421	1.872
Education_c × SII‐c	−0.120	0.093	1.661	1	0.198	0.886	0.738	1.065
Depression, yes = 1	0.479	0.302	2.511	1	0.113	1.614	0.893	2.919
FrSBe premorbid apathy	0.040	0.006	39.353	1	< 0.001	1.040	1.028	1.053
Constant	−3.718	0.958	15.076	1	< 0.001	0.024		

*Note*: Theory‐driven hierarchical forced‐entry binary logistic regression (all predictors entered simultaneously). Bold red *p* values indicate *p* < 0.05. Depression coded 1 = history of affective disorder. Marital status coded 1 = single/unpartnered. Sex coded 1 = female. Education × SII = interaction between years of education and SII. Predictors entered in 5 prespecified blocks: (1) age, sex; (2) disease duration, ALS‐FRS‐R total score; (3) education, occupation level; (4) SII_c, marital status, Education_c × SII_c; and (5) premorbid FrSBe subscale score, depression history. All blocks used forced entry. Premorbid FrSBe apathy = Family Form “before” apathy subscale. Apathy impairment criterion: subscale score ≥ 65 (for details see the text). ALS = amyotrophic lateral sclerosis; ALSFRS‐R = ALS Functional Rating Scale‐Revised; df = degree of freedom; Exp(B) = odds ratio; FrSBe = Frontal Systems Behavior Scale; SII = Social Interaction Index.

**TABLE 4:** Final Model: FrSBe Disinhibition Subscale – Binary Logistic Regression

Variables	*B*	SE	Wald	df	*p*‐value	Exp(B)	95% Confidence Interval Exp(B)	
							Lower	Upper
Sex, F = 1	−0.454	0.212	4.584	1	0.032	0.635	0.419	0.962
Age	0.012	0.011	1.105	1	0.293	1.012	0.990	1.034
ALSFRS‐R total score	−0.210	0.208	1.017	1	0.313	0.811	0.539	1.219
Disease duration, mo	0.002	0.009	0.039	1	0.843	1.002	0.984	1.020
Education_c, yr	0.003	0.030	0.010	1	0.921	1.003	0.945	1.064
Occupation level	−0.124	0.165	0.564	1	0.453	0.884	0.640	1.220
SII_c	0.605	0.732	0.683	1	0.409	1.831	0.436	7.694
Marital status, single = 1	−0.735	0.746	0.970	1	0.325	0.480	0.111	2.070
Education_c × SII‐c	−0.280	0.129	4.691	1	0.030	0.756	0.586	0.974
Depression, yes = 1	0.411	0.372	1.216	1	0.270	1.508	0.727	3.128
FrSBe premorbid disinhibition	0.051	0.008	45.274	1	< 0.001	1.052	1.037	1.068
Constant	−6.500	1.339	23.580	1	< 0.001	< .001		

*Note*: Theory‐driven hierarchical forced‐entry binary logistic regression (all predictors entered simultaneously). Bold red *p* values indicate *p* < 0.05. Depression coded 1 = history of affective disorder. Marital status coded 1 = single/unpartnered. Sex coded 1 = female. Education × SII = interaction between years of education and SII. Predictors entered in 5 prespecified blocks: (1) age, sex; (2) disease duration, ALS‐FRS‐R total score; (3) education, occupation level; (4) SII_c, marital status, Education_c × SII_c; and (5) premorbid FrSBe subscale score, depression history. All blocks used forced entry. Premorbid FrSBe disinhibition = Family Form “before” disinhibition subscale. Disinhibition impairment criterion: subscale score ≥ 65 (for details see the text). ALS = amyotrophic lateral sclerosis; ALSFRS‐R = ALS Functional Rating Scale‐Revised; df = degree of freedom; Exp(B) = odds ratio; FrSBe = Frontal Systems Behavior Scale; SII = Social Interaction Index.

The Education_c × SII_c interaction was significant in the total score (*p* = 0.025, Exp(B) = 0.801), disinhibition (*p* = 0.030, Exp(B) = 0.756), and executive dysfunction models (*p* = 0.038, Exp(B) = 0.776), consistently protective, but absent for FrSBe apathy. The interaction coefficient was algebraically invariant to mean‐centering and identical to the uncentred solution, indicating that the interaction term was not driven by centering or scaling choices that the synergistic effect is not an artifact of scaling. Education_c and SII_c considered alone did not reach significance in any model indicating that the protective influence of education and social engagement operates synergistically rather than additively. Age was a robust predictor for total score (Exp(B) = 1.027, *p* = 0.002), apathy (Exp(B) = 1.020, *p* = 0.009), and executive dysfunction (Exp(B) = 1.048, *p* < 0.001) but entirely absent for disinhibition. A history of affective disorder was a domain‐specific risk factor, significant exclusively for executive dysfunction (*p* = < 0.001, Exp(B) = 3.187), indicating that a premorbid history of depression or bipolar disorder is associated with approximately 3 times the odds of post‐disease executive impairment; it did not reach significance for total score (*p* = 0.132) or apathy (*p* = 0.113). Female sex was associated with lower odds of disinhibition (Exp(B) = 0.635, *p* = 0.032). Occupation level was protective only for executive dysfunction (Exp(B) = 0.697, *p* = 0.018), possibly reflecting career‐specific maintenance of planning and organizational functions. Full results for the final model of each analysis are presented in Tables 2, 3, 4, 5. The pattern of significant predictors across all FrSBe and ECAS‐CI models, with ORs and 95% confidence intervals, is displayed in Figure 1.

**TABLE 5:** Final Model: FrSBe Executive Dysfunction Subscale – Binary Logistic Regression

Variable	*B*	SE	Wald	df	*p*‐value	Exp(B)	95% Confidence Interval Exp(B)	
							Lower	Upper
Sex, F = 1	0.279	0.210	1.765	1	0.184	1.322	0.876	1.996
Age	0.047	0.011	17.150	1	< 0.001	1.048	1.025	1.072
ALSFRS‐R total score	−0.184	−0.207	0.797	1	0.373	0.832	0.555	1.247
Disease duration, mo	−0.010	0.010	1.050	1	0.305	0.990	0.971	1.009
Education_c, yr	0.050	0.029	3.022	1	0.082	1.052	0.994	1.113
Occupation level	−0.361	0.153	5.601	1	0.018	0.697	0.517	0.940
SII_c	−0.750	0.657	1.301	1	0.254	0.472	0.130	1.714
Marital status, single = 1	−1.706	1.034	2.722	1	0.099	0.182	0.024	1.378
Education_c × SII‐c	−0.253	0.122	4.308	1	0.038	0.776	0.611	0.986
Depression, yes = 1	1.159	0.334	12.033	1	< 0.001	3.187	1.656	6.135
FrSBe premorbid executive	0.050	0.008	37.885	1	< 0.001	1.052	1.035	1.069
Constant	−6.829	1.260	29.365	1	< 0.001	0.001		

*Note*. Theory‐driven hierarchical forced‐entry binary logistic regression (all predictors entered simultaneously). Bold red *p* values indicate *p* < 0.05. Depression coded 1 = history of affective disorder. Marital status coded 1 = single/unpartnered. Sex coded 1 = female. Education × Social int. index = interaction between years of education and Social Interaction Index. Predictors entered in five pre‐specified blocks: (1) age, sex; (2) disease duration, ALS‐FRS‐R total score; (3) education, occupation level; (4) SII_c, marital status, Education_c × SII_c; (5) premorbid FrSBe subscale score, depression history. All blocks used forced entry. Premorbid FrSBe executive = Family Form “before” executive dysfunction subscale. Executive dysfunction impairment criterion: subscale score ≥ 65 (for details see the text).

ALS = amyotrophic lateral sclerosis; ALSFRS‐R = ALS Functional Rating Scale‐Revised; df = degree of freedom; Exp(B) = odds ratio; FrSBe = Frontal Systems Behavior Scale; SII = Social Interaction Index.

**Figure 1:**
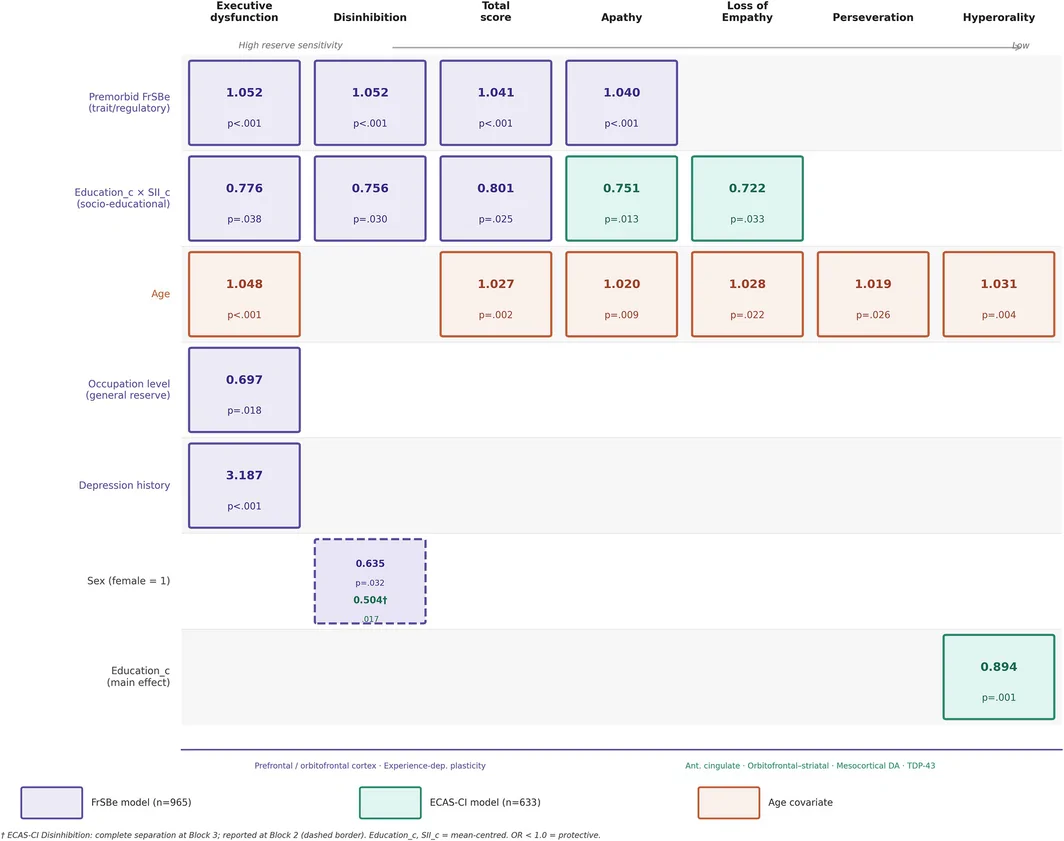
Reserve‐sensitivity gradient across behavioral domains in ALS. Matrix displaying the pattern of significant BvR predictors across 6 behavioral domains, ordered from high (left) to low (right) reserve sensitivity. Rows represent BvR components entered in the hierarchical models; columns represent behavioral outcome domains. Filled cells indicate a significant predictor in the final forced‐entry model, with the corresponding OR and *p* value shown. Empty cells indicate nonsignificant effects. Color denotes instrument and model type: purple = significant in FrSBe confirmatory models (n = 965); teal = significant in ECAS‐CI exploratory models (n = 633); coral = age effect only (biologically determined domains). The dashed‐border cell for perseveration age indicates a significant predictor‐level effect (*p* = 0.026) within a nonsignificant overall model (*p* = 0.082). Annotated bars at the bottom map each domain to its primary neurobiological substrate. n/a = no premorbid FrSBe assessment available for ECAS‐CI models. The ECAS‐CI disinhibition model is not shown (reported at block 2 only; see Supplementary Table S3). ALS = amyotrophic lateral sclerosis; BvR = behavioral reserve; ECAS‐CI = Edinburgh Cognitive and Behavioural ALS Screen–Carer Interview; FrSBe = Frontal Systems Behavior Scale; OR = odds ratio from theory‐driven hierarchical forced‐entry binary logistic regression. [Color figure can be viewed at www.annalsofneurology.org]

### Sensitivity Analyses

To further address the concern of informant‐based measurement dependence, 3 prespecified sensitivity analyses were conducted: exclusion of borderline premorbid scores (SA1, n = 911), exclusion of cases with implausibly large before–after discrepancies exceeding the 95th percentile of the distribution (SA2, n = 740), and both exclusions combined (SA3, n = 721). Across all 3 restricted samples, the premorbid FrSBe effect was stable or numerically larger than in the primary analysis (ORs = 1.045–1.076, all *p* < 0.001; Supplementary Table S1). Critically, effect sizes were largest in SA2 and SA3, the samples with the most restricted rating variability, the opposite of the pattern expected under an informant halo effect, which would predict attenuation rather than amplification when cases with large discrepancies are removed. This amplification pattern is consistent with a genuine signal that is partially attenuated by measurement noise in the full sample and is less consistent with systematic informant bias as the sole explanation.

### ECAS‐CI Models: Cross‐Instrument Replication and Domain‐Specific Sensitivity

In the absence of a premorbid baseline, the ECAS‐CI models were sparser but revealed the robustness of the socio‐educational reserve mechanism. The Education_c × SII_c interaction reached significance in 2 of 5 subscale models: apathy/inertia (*p* = 0.013, Exp(B) = 0.751; Supplementary Table S3) and loss of empathy (*p* = 0.033, Exp(B) = 0.722; Supplementary Table S5). Education_c emerged as a significant independent predictor of hyperorality (OR = 0.894, *p* = 0.001; Supplementary Table S7). The ECAS‐CI total score model showed no significant reserve predictors (Supplementary Table S2), underscoring the amplifying role of premorbid baseline measurement in rendering reserve effects detectable.

Perseveration was the most reserve‐resistant domain. In the ECAS‐CI perseveration model, all reserve‐related predictors were nonsignificant; age was the only variable reaching individual‐level significance (Exp(B) = 1.019, *p* = 0.026; see Supplementary Table S5). The ECAS‐CI disinhibition model is reported at block 2 due to complete separation caused by marital status in block 3 (coefficient = −18.9, SE = 8,297); the dominant predictor at block 2 was female sex (Exp(B) = 0.504, *p* = 0.017; see Supplementary Table S3), indicating that female sex is protective for disinhibition. Full results for the final model of each ECAS‐CI analysis are presented in Supplementary Tables S2 to S7. Discriminative accuracy was consistently higher in FrSBe models than in ECAS‐CI models (area under the curve [AUC] range = 0.670–0.751 vs 0.557–0.679), and model fit followed the same pattern (Nagelkerke *R* ^2^ range = 0.025–0.169 vs 0.027–0.103). Model fit and discriminative accuracy indices for all 10 models are reported in Supplementary Table S8. The full pattern of domain‐specific reserve effects across the ALS‐FTD spectrum is illustrated in Figure 2.

**Figure 2:**
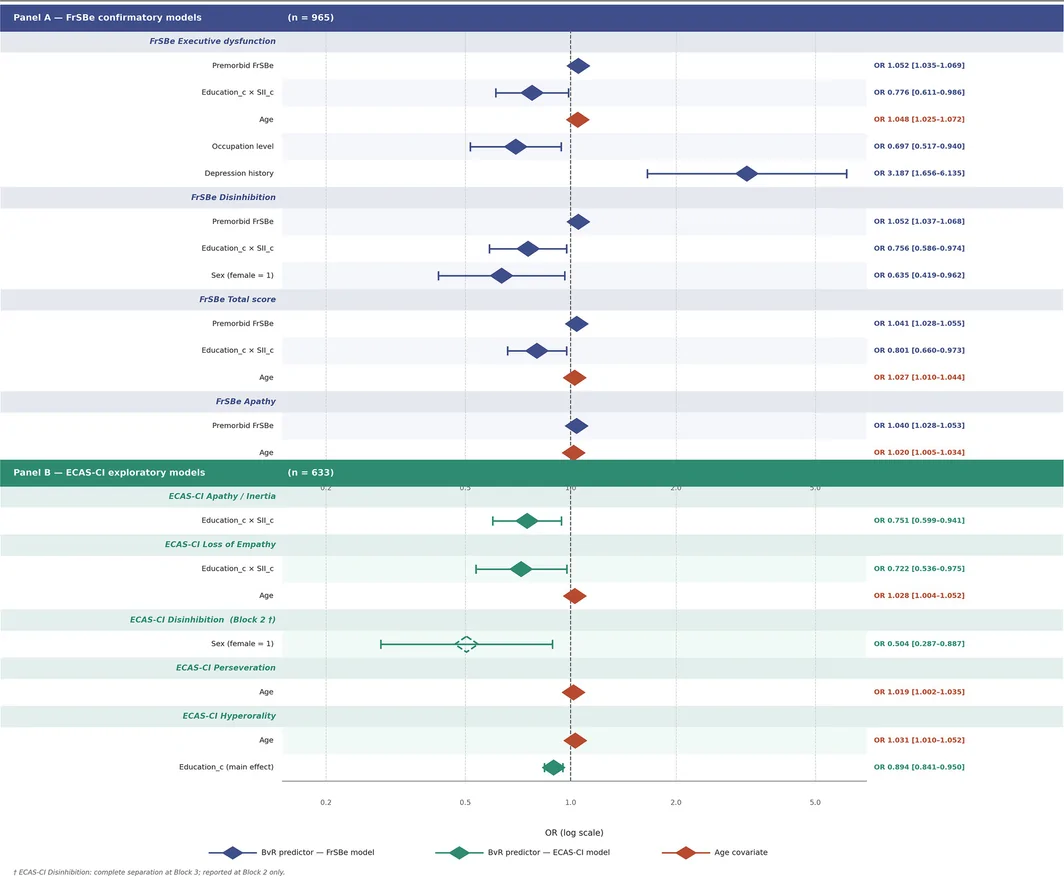
Forest plot of significant predictors across FrSBe and ECAS‐CI models. Odds ratios (ORs) with 95% confidence intervals for all statistically significant predictors in the final forced‐entry binary logistic regression models. Each row represents one predictor–outcome combination. Panels are grouped by outcome instruments: upper panel, FrSBe confirmatory models (n = 965); lower panel, ECAS‐CI exploratory models (n = 633). The vertical reference line is set at OR = 1.0 (no effect). Open diamond = block 2 result (ECAS‐CI disinhibition; complete separation at block 3). For the premorbid FrSBe trait component, OR values represent per‐unit increases in the subscale score; ORs >1.0 indicate increased odds of impairment with higher premorbid scores. For the Education_c × SII_c interaction and occupation level, OR <1.0 reflects a protective effect. For sex (female coded 1), OR <1.0 indicates lower odds of impairment in female patients. For age, OR >1.0 reflects increased odds per year of age. Confidence intervals that do not cross 1.0 confirm statistical significance. ECAS‐CI = Edinburgh Cognitive and Behavioural ALS Screen Carer Interview; FrSBe = Frontal Systems Behavior Scale; OR = odds ratio; SII = Social Interaction Index. [Color figure can be viewed at www.annalsofneurology.org]

## Discussion

This study provides the first large‐scale empirical study for a BvR framework in ALS, showing that behavioral heterogeneity is systematically shaped by premorbid regulatory capacity, socio‐educational exposure, and biological modifiers, supporting the plausibility of BvR as a potentially distinct construct within frontolimbic systems. Prior works have shown that CR in ALS primarily modulates cognitive performance and disease trajectory^7^, ^8^, ^9^ but exerts limited or indirect effects on behavioral symptoms.^7^, ^9^ Our results identify a complementary, cognitively independent mechanism directly shaping behavioral resilience.

The dominant role of premorbid behavioral regulation across all FrSBe models represents the central finding of this study. Premorbid domain‐specific FrSBe scores consistently emerged as the strongest predictors of post‐disease impairment. This suggests that the trait/regulatory component may represent a central contributor within the proposed BvR model, indicating that baseline differences in impulse control, motivation, and executive self‐regulation critically shape vulnerability to behavioral decompensation. Our findings extend neuroimaging‐based evidence of BvR in bvFTD,^14^ by demonstrating that informant‐derived premorbid behavioral measures provide a clinically accessible proxy for the same construct. The progressive increase in effect size across domains is consistent with the principle that reserve operates most strongly at the level of specific functional systems, in line with findings in FTD.^4^, ^5^ The use of domain‐specific premorbid scores warrants methodological comment. A potential concern is that “before” and “after” scores share informant and instrument; however, 3 considerations argue against measurement dependence: the 2 forms are conceptually and temporally distinct; impairment is defined by post‐disease thresholds independently of the premorbid score; and the domain‐specificity of effects (premorbid executive scores predicting executive impairment and premorbid disinhibition scores predicting disinhibitory impairment) is precisely what a genuine reserve construct predicts. Prespecified sensitivity analyses further supported the robustness of the premorbid FrSBe effect, with amplification of effect sizes in the most restricted subsamples.

A second major finding is the robustness of the Education_c × SII_c interaction, replicated across multiple domains and instruments. Intellectual enrichment and sustained social complexity act synergistically to confer resilience, building on evidence that occupational profiles emphasizing social interaction modulate neural vulnerability in bvFTD^13^ and aligns with broader literature indicating that reserve is not reducible to single proxies such as education alone.^6^, ^31^, ^32^ The consistency of effect directions across models suggests a reproducible statistical association across behavioral domains. The absence of significant main effects for education and SII further supports this interpretation: neither variable confers independent additive protection at average levels of the other; their protective influence is conditional on their co‐occurrence and operates exclusively through synergistic interaction. Because this was an observational cross‐sectional study, these associations should not be interpreted as demonstrating causal effects of socio‐educational exposures, but rather as identifying candidate reserve‐related correlates.

The neurobiological interpretation is that sustained engagement in cognitively and socially demanding environments may contribute to maintenance or efficiency of frontolimbic networks subserving behavioral regulation, including the salience network and medial frontal systems implicated in interoception, emotional regulation, and social cognition.^10^, ^11^, ^31^

A key contribution of this study is the demonstration of domain‐specific variability in reserve effects. Executive dysfunction and disinhibition were the most strongly modulated domains, whereas apathy and perseveration were relatively resistant. This gradient is neurobiologically coherent. Executive and disinhibitory functions depend on prefrontal and orbitofrontal networks with high experience‐dependent plasticity, making them more susceptible to reserve effects.^10^, ^11^ In contrast, apathy is closely linked to anterior cingulate and mesocortical dopaminergic systems,^33^ whereas perseveration reflects orbitofronto‐striatal circuitry driven more directly by TDP‐43 pathology.^34^ These systems may be comparatively less sensitive to reserve‐related lifetime exposures, explaining their relative resistance. This domain‐specificity reinforces the need to conceptualize reserve as a functionally heterogeneous phenomenon rather than a unitary construct. A schematic overview of the 4‐component BvR model and its proposed pathways to domain‐specific behavioral outcomes, including the reserve‐sensitivity gradient, is presented in Figure 3.

**Figure 3:**
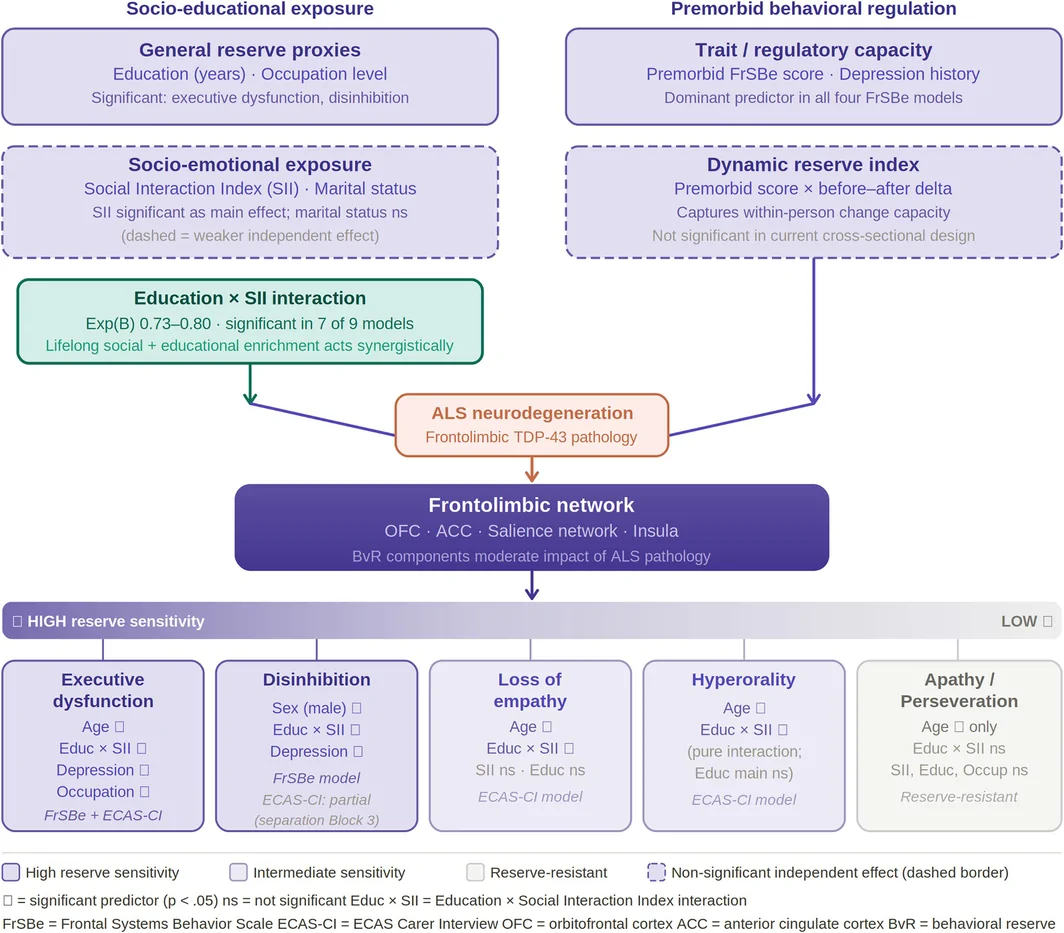
Conceptual framework of behavioral reserve in ALS. Schematic illustrating the four‐component BvR model and its proposed pathways to domain‐specific behavioral outcomes. The 4 components (described in detail in the Methods section) jointly moderate the impact of ALS‐related frontolimbic neurodegeneration on domain‐specific behavioral outcomes, with effect magnitude varying along the reserve‐sensitivity gradient (executive dysfunction and disinhibition most modulated; apathy and perseveration least). Dashed outlines indicate components whose independent effects were nonsignificant in the current analyses. ALS = amyotrophic lateral sclerosis; BvR, behavioral reserve; FrSBe, Frontal Systems Behavior Scale; SII, Social Interaction Index. [Color figure can be viewed at www.annalsofneurology.org]

The comparison between FrSBe and ECAS‐CI models provides important methodological insight. The ECAS‐CI models were treated as exploratory given their smaller sample, cross‐sectional measurement structure, and shorter observation window. Accordingly, statistically significant findings from ECAS‐CI subscale models should be considered hypothesis‐generating until replicated in independent cohorts with prospectively harmonized behavioral assessment. Within this framing, the replication of the Education_c × SII_c interaction across 2 ECAS‐CI subscales (apathy/inertia and loss of empathy) is noteworthy precisely because it was not expected to be detectable without a premorbid baseline, and its emergence despite this limitation strengthens confidence in the socio‐educational reserve mechanism. More broadly, models incorporating premorbid baseline measures consistently demonstrated greater sensitivity to reserve effects, whereas those based on cross‐sectional symptom thresholds yielded sparser results. This observation aligns with methodological concerns regarding reserve measurement: proxy‐based approaches provide indirect estimates, whereas residual methods may introduce bias when predictors correlate with outcomes.^18^, ^34^, ^35^ Instruments capturing change from baseline offer a direct means of modeling the trait component of reserve, which appears essential for rendering BvR effects detectable in confirmatory analyses.

Our findings also clarify the relationship between behavioral and CR in ALS. Simão et al^7^ demonstrated that CR influences behavioral symptoms indirectly through cognitive performance, whereas Temp et al^9^ showed that standard CR proxies do not reduce the risk of behavioral impairment. Aiello et al^8^ further demonstrated that CR effects are domain‐specific, primarily affecting executive function. Complementarily, Hermann et al^15^ showed using neuroimaging that cognitively normal patients with ALS display younger brain age than healthy controls, suggesting a structural cerebral reserve specifically in the absence of cognitive‐behavioral impairment. The present results resolve this apparent dissociation by demonstrating that behavioral resilience depends on distinct mechanisms, specifically premorbid regulatory capacity and socio‐emotional experience. Thus, CR and BvR should be considered integrated but dissociable constructs, operating on distinct neural systems and clinical domains.^36^

Additional findings provide convergent support for this framework. The domain‐specific effect of depression history, particularly its exclusive association with executive dysfunction, and its absence for total score and apathy, is consistent with the selective overlap between affective disorders and dorsolateral prefrontal–anterior cingulate circuitry. ALS‐related apathy is neurobiologically distinct from depressive apathy, being more directly linked to anterior cingulate degeneration, and the forced‐entry model correctly separates these 2 constructs by showing that affective history does not independently predict motivational impairment once all other predictors are held constant. The effect of depression history on executive dysfunction indicates that a premorbid affective disorder approximately triples the odds of post‐disease executive impairment, consistent with the shared vulnerability of dorsolateral prefrontal networks in both conditions. The selective protective effect of occupation level for executive dysfunction further supports the role of occupational cognitive demands in maintaining prefrontal systems.^32^ The protective effect of female sex for disinhibition was replicated across both instruments (FrSBe: Exp(B) = 0.635, *p* = 0.032; ECAS‐CI: Exp(B) = 0.504, *p* = 0.017), extending evidence from FTD^37^, ^38^ and underscoring the biological robustness of sex differences in orbitofrontal inhibitory control across neurodegenerative conditions. Finaly, the domain‐specific effect for apathy of bulbar onset is in line with the observation of a more frequent cognitive impairment in patients with bulbar onset.^7^, ^39^

These results have direct clinical implications. First, they may help future risk stratification models by identifying individuals with low premorbid regulatory capacity and limited socio‐educational exposure as those at highest risk for behavioral involvement. Second, they suggest that some factors associated with the proposed BvR construct may be modifiable. The consistent interaction between education and social exposure indicates that interventions targeting socially and cognitively enriching activities may enhance resilience. Evidence from other cohorts suggests that multidomain interventions produce synergistic benefits,^17^ raising the possibility that similar approaches could be adapted for ALS, particularly in early and premorbid disease stages.

Several limitations should be considered. The cross‐sectional design of this study precludes direct assessment of longitudinal trajectories and reserve‐related differences in rate of decline. Motor severity (ALSFRS‐R) and disease duration were included as clinical covariates in all models; neither reached significance, arguing against confounding by disease stage. The ECAS‐CI cohort was smaller, likely to reduce statistical power. No correction for multiple comparisons was applied across the 10 models; whereas the prespecified confirmatory/exploratory distinction mitigates this concern for the primary outcomes, the possibility of type I error inflation in the exploratory ECAS‐CI analyses cannot be excluded. The study population derived from a single specialist center, potentially limiting generalizability. External validation in multicenter cohorts will be important to determine transportability of the proposed BvR framework across health care systems and sociocultural contexts. In addition, our framework did not incorporate neighborhood‐level socioeconomic factors, which have been shown to influence disease progression in bvFTD^16^ and may represent an additional dimension of reserve.

This study supports BvR as a useful multidimensional framework for understanding behavioral heterogeneity in ALS, distinct from but complementary to CR. Premorbid regulatory capacity emerged as the strongest associated factor, whereas the interaction between educational attainment and socioemotional exposure provides a robust and potentially modifiable mechanism of resilience. These findings extend reserve theory beyond cognition, provide a framework for understanding behavioral heterogeneity in ALS, and identify targets for risk stratification and future intervention strategies.

## Author Contributions

F.P., B.I., C.M., U.M., E.Ma., S.C., G.P., E.Mi., V.P., R.V., G.M., F.D., A.C., A.Ca., and A.Ch. contributed to the conception and design of the study; F.P., B.I., C.M., U.M., E.Ma., S.C., G.P., E.Mi., V.P., R.V., G.M., F.D., A.C., A.Ca., and A.Ch. contributed to the acquisition and analysis of data; F.P., B.I., G.M., .A.Ca., and A.Ch. contributed to drafting the text or preparing the figures.

## Potential Conflicts of Interest

The authors declare no conflicts of interest.

## Supporting information


**Supplementary Figure S1.** Robustness of the premorbid FrSBe effect across prespecified sensitivity analyses. ORs with 95% confidence intervals for the domain‐specific premorbid FrSBe score as a predictor of post‐disease behavioral impairment, shown for the 3 confirmatory FrSBe models in which this predictor was entered: (A) FrSBe total score, (B) disinhibition, and (C) executive dysfunction. Within each panel, estimates are plotted for the primary analysis (full sample) and for the 3 restricted samples: SA1, exclusion of borderline premorbid scores (n = 911); SA2, exclusion of cases with before–after discrepancies exceeding the 95th percentile of the distribution (n = 740); and SA3, both exclusions combined (n = 721). The primary‐analysis sample sizes were n = 965 (total), n = 965 (disinhibition), and n = 633 (executive dysfunction). Across all restricted samples, the premorbid FrSBe effect remained significant (all *p* < 0.001) and was stable or numerically larger than in the primary analysis (ORs = 1.045–1.076), with the largest effect sizes observed in SA2 and SA3 – the samples with the most restricted rating variability. This amplification in the most restricted subsamples is the opposite of the attenuation expected under an informant halo effect, and is consistent with a genuine premorbid signal partially masked by measurement noise in the full sample. Each marker denotes the point estimate (OR) and horizontal bars the 95% confidence interval; the vertical reference line marks OR = 1.0, and confidence intervals that do not cross it indicate statistical significance. FrSBe = Frontal Systems Behavior Scale; OR = odds ratio; SA = sensitivity analysis.

**Supplementary Table S1.** Sensitivity analyses. Effect of premorbid FrSBe total score on FrSBe total score impairment across 3 restricted samples. FrSBe = Frontal Systems Behavior Scale.
**Supplementary Table S2.** Final model: ECAS‐CI Total behavioural score – binary logistic regression. ECAS‐CI = Edinburgh Cognitive and Behavioural ALS Screen–Carer Interview.
**Supplementary Table S3.** Final model: ECAS‐CI Apathy/Inertia subscale – binary logistic regression. ECAS‐CI = Edinburgh Cognitive and Behavioural ALS Screen–Carer Interview.
**Supplementary Table S4.** Final model: ECAS‐CI Disinhibition subscale – binary logistic regression (Block 2). ECAS‐CI = Edinburgh Cognitive and Behavioural ALS Screen–Carer Interview.
**Supplementary Table S5.** Final model: ECAS‐CI Loss of Empathy subscale – binary logistic regression. ECAS‐CI = Edinburgh Cognitive and Behavioural ALS Screen–Carer Interview.
**Supplementary Table S6.** Final model: ECAS‐CI Perseverative/Compulsive Behavior subscale – binary logistic regression. ECAS‐CI = Edinburgh Cognitive and Behavioural ALS Screen–Carer Interview.
**Supplementary Table S7.** Final model: ECAS‐CI Hyperorality/Dietary Changes subscale – binary logistic regression. ECAS‐CI = Edinburgh Cognitive and Behavioural ALS Screen–Carer Interview.
**Supplementary Table S8.** Model fit and discriminative accuracy indices for all 10 hierarchical logistic regression models.

## Data Availability

Anonymized data relating to this article will be made available by request from any qualified investigator, subject to approval from the Comitato Etico Azienda Ospedaliero‐Universitaria Città della Salute e della Scienza, Torino, Italy, to the corresponding authors.
