# Supplementary material for: Toward a Behavioral Reserve Model in Amyotrophic Lateral Sclerosis
Source: Ann Neurol. 2026 Jul 30;100(5):934–47. doi: 10.1002/ana.78315 (PMC13688070; doi:10.1002/ana.78315)
Supplement: Supplementary file 1 [file ANA-100-934-s001.docx]

**Table S1.** *Sensitivity analyses: effect of premorbid FrSBe total score on FrSBe total score impairment across three restricted samples*

| **Sample** | **Exclusion criterion** | **n** | **OR** | **95% CI** | **p-value** |
| --- | --- | --- | --- | --- | --- |
| **Primary analysis** | None | 965 | 1.041 | 1.028–1.053 | < .001 |
| **SA1** | Borderline premorbid FrSBe excluded (T-score 60–64; n excluded = 54) | 911 | 1.045 | 1.032–1.059 | < .001 |
| **SA2** | High before–after discrepancy excluded (delta > 95th percentile [≥ 29 points]; n excluded = 184) | 781 | 1.076 | 1.045–1.108 | < .001 |
| **SA3** | Both exclusions combined (borderline premorbid + high discrepancy; n excluded = 225) | 740 | 1.072 | 1.044–1.100 | < .001 |

Note*.* All analyses used the same theory-driven hierarchical forced-entry binary logistic regression with four pre-specified blocks as the primary analysis. OR = odds ratio for the premorbid FrSBe total score (Family Form ‘before’) in the final model (Block 4), adjusted for age, sex, education, occupation level, Social Interaction Index, Education × SII interaction, and marital status. 95% CI = confidence interval. SA1 excludes patients with borderline premorbid FrSBe total T-scores (60–64), who do not meet strict criteria for frontal systems normality. SA2 excludes patients whose before–after delta exceeded the 95th percentile of the distribution (≥29 T-score points), flagging potential informant rating inconsistency. SA3 applies both exclusions simultaneously. Effect sizes are numerically larger in restricted samples, consistent with attenuation of the premorbid signal by measurement noise in the full sample.

**Table S2.** *Final model: ECAS-CI Total behavioural score — binary logistic regression*

| **Variable** | **B** | **S.E.** | **Wald** | **df** | **Sign.** | **Exp(B)** | **95% CI Exp(B)** | |
| --- | --- | --- | --- | --- | --- | --- | --- | --- |
|  |  |  |  |  |  |  | **Lower** | **Upper** |
| Sex (female = 1) | -.215 | .169 | 1.619 | 1 | .203 | 0.806 | .579 | 1.728 |
| Age | .014 | .008 | 3.328 | 1 | .068 | 1.014 | .999 | 1.010 |
| Education_c (years) | -.037 | .024 | 2.406 | 1 | .121 | .964 | .920 | 1.010 |
| ALSFRS-R | .329 | .225 | 2.139 | 1 | .144 | 1.389 | .894 | 2.159 |
| Occupation level | -.093 | .122 | .584 | 1 | .445 | .911 | .717 | 1.157 |
| Time from onset to testing (months) | .005 | .007 | .551 | 1 | .458 | 1.005 | .992 | 1.019 |
| Site of onset (bulbar = 1) | .004 | .193 | .000 | 1 | .983 | 1.004 | .989 | 1.018 |
| SII_c | -.736 | .556 | 1.753 | 1 | .186 | .479 | .161 | 1.424 |
| Marital status (single = 1) | -.328 | .459 | .510 | 1 | .475 | .721 | .293 | 1.771 |
| Education_c × SII_c | .099 | .111 | .795 | 1 | .373 | 1.104 | .888 | 1.371 |
| Depression (yes = 1) | .262 | .368 | .509 | 1 | .475 | 1.300 | .632 | 2.672 |
| Constant | .522 | 1.022 | 0.261 | 1 | .609 | 1.685 |  |  |
|  | | | | | | | | |

Note. Theory-driven hierarchical forced-entry binary logistic regression. Predictors entered in five pre-specified blocks: (1) age, sex; (2) clinical covariates (disease duration in months, ALS Functional Rating Scale-Revised [ALSFRS-R] total score at time of testing); Site of onset; (3) Education_c, occupation level; (4) Social Interaction Index, marital status, Education_c × SII_c; (5) depression history. Bold red p-values indicate p < .05. Exp(B) = odds ratio; CI = confidence interval. Site of onset coded 1= bulbar; Sex coded 1 = female. Depression coded 1 = history of affective disorder (yes). Marital status coded 1 = partnered. Education_c × SII_c = interaction between mean-centred Education_c and mean-centred SII_c. No premorbid assessment available for ECAS-CI models. ECAS-CI Total impairment criterion: any subscale meeting clinical threshold. No reserve predictor reached significance in the final model.

**Table S3.** *Final model: ECAS-CI Apathy/Inertia subscale — binary logistic regression*

| **Variable** | **B** | **S.E.** | **Wald** | **df** | **Sign.** | **Exp(B)** | **95% CI Exp(B)** | |
| --- | --- | --- | --- | --- | --- | --- | --- | --- |
|  |  |  |  |  |  |  | **Lower** | **Upper** |
| Sex (female = 1) | .108 | .179 | .363 | 1 | .547 | 0.897 | .633 | 1.276 |
| Age | .005 | .008 | .368 | 1 | .544 | 1.005 | .989 | 1.022 |
| Education_c (years) | -.033 | .026 | 1.669 | 1 | .196 | .967 | .920 | 1.017 |
| Occupation level | -.158 | .130 | 1.482 | 1 | .223 | .854 | .661 | 1.101 |
| ALSFRS-R | .042 | -.220 | .037 | 1 | .847 | .958 | .622 | 1.476 |
| Time from onset to testing (months) | -.001 | .007 | .005 | 1 | .943 | .999 | .985 | 1.014 |
| Site of onset (bulbar = 1) | .510 | .201 | 6.460 | 1 | **.011** | 1.665 | 1.112 | 2.468 |
| SII_c | .085 | .592 | .021 | 1 | .886 | 1.088 | .341 | 3.472 |
| Marital status (single = 1) | -.270 | .496 | .296 | 1 | .586 | .764 | .289 | 2.017 |
| Education_c × SII_c | -.287 | .115 | 6.171 | 1 | **.013** | .751 | .599 | .941 |
| Depression (yes = 1) | .459 | .371 | 1.527 | 1 | .217 | 1.582 | .764 | 3.275 |
| Constant | -1.572 | 1.074 | 2.141 | 1 | .143 | .208 |  |  |
|  | | | | | | | | |

Note. Theory-driven hierarchical forced-entry binary logistic regression. Predictors entered in five pre-specified blocks: (1) age, sex; (2) clinical covariates (disease duration in months, ALS Functional Rating Scale-Revised [ALSFRS-R] total score at time of testing, site of onset); (3) Education_c, occupation level; (4) Social Interaction Index, marital status, Education_c × SII_c; (5) depression history. Bold red p-values indicate p < .05. Exp(B) = odds ratio; CI = confidence interval. Site of onset coded 1 = bulbar; Sex coded 1 = female. Depression coded 1 = history of affective disorder (yes). Marital status coded 1 = partnered. Education_c × SII_c = interaction between mean-centered. Education_c and mean-centred SII_c. No premorbid assessment available for ECAS-CI models. ECAS-CI Apathy/Inertia impairment criterion: subscale meeting clinical threshold.

**Table S4.** *Final model: ECAS-CI Disinhibition subscale — binary logistic regression (Block 2)*

| **Variable** | **B** | **S.E.** | **Wald** | **df** | **Sign.** | **Exp(B)** | **95% CI Exp(B)** | |
| --- | --- | --- | --- | --- | --- | --- | --- | --- |
|  |  |  |  |  |  |  | **Lower** | **Upper** |
| Sex (female = 1) | .685 | .288 | 5.650 | 1 | **.017** | 0.504 | 0.287 | 0.887 |
| Age | .015 | .013 | 1.397 | 1 | .237 | 1.015 | .990 | 1.041 |
| Education_c (years) | -.036 | .039 | .878 | 1 | .349 | .964 | .894 | 1.040 |
| ALSFRS-R | -.174 | .381 | .208 | 1 | .352 | 1.190 | .563 | 2.513 |
| Site of onset (bulbar = 1) | .158 | .379 | .174 | 1 | .677 | 1.171 | .557 | 2.462 |
| Time from onset to testing (months) | -.012 | .013 | .865 | 1 | .352 | .988 | .963 | 1.014 |
| Occupation level | .170 | .187 | .819 | 1 | .366 | 1.185 | .821 | 1.711 |
| Constant | -3.617 | 1.032 | 12.280 | 1 | <.001 | .027 |  |  |
|  | | | | | | | | |

Note. Theory-driven hierarchical forced-entry binary logistic regression. Predictors entered in five pre-specified blocks: (1) age, sex; (2) clinical covariates (disease duration in months, ALS Functional Rating Scale-Revised [ALSFRS-R] total score at time of testing; site of onset); (3) Education_c, occupation level; (4) Social Interaction Index, marital status, Education_c × SII_c; (5) depression history. Bold red p-values indicate p < .05. Exp(B) = odds ratio; CI = confidence interval. Site of onset coded 1 = bulbar. Sex coded 1 = female. No premorbid assessment available for ECAS-CI models. ECAS-CI Disinhibition impairment criterion: subscale meeting clinical threshold. Model reported at Block 3 (demographics + clinical + general reserve proxies): complete separation occurred when marital status was entered in Block 4 (coefficient −18.9, SE 8297), rendering Blocks 4 and 5 unreliable.

**Table S5.** *Final model: ECAS-CI Loss of Empathy subscale — binary logistic regression*

| **Variable** | **B** | **S.E.** | **Wald** | **df** | **Sign.** | **Exp(B)** | **95% CI Exp(B)** | |
| --- | --- | --- | --- | --- | --- | --- | --- | --- |
|  |  |  |  |  |  |  | **Lower** | **Upper** |
| Sex (female = 1) | .270 | .250 | 1.165 | 1 | .280 | 0.764 | .468 | 1.245 |
| Age | .028 | .012 | 5.267 | 1 | **.022** | 1.028 | 1.004 | 1.052 |
| Education_c (years) | .048 | .035 | 1.941 | 1 | .164 | 1.050 | .980 | 1.124 |
| Occupation level | -.104 | .181 | .329 | 1 | .566 | .902 | .633 | 1.285 |
| ALSFRS-R | .521 | .446 | 1.363 | 1 | .243 | 1.683 | .702 | 4.033 |
| Time from onset to testing (months) | -.012 | .012 | 1.145 | 1 | .285 | .988 | .966 | 1.010 |
| Site of onset (bulbar = 1) | .649 | .352 | 3.405 | 1 | .065 | 1.916 | .961 | 3.817 |
| SII_c | .970 | .888 | 1.191 | 1 | .275 | 2.637 | .462 | 15.038 |
| Marital status (single = 1) | .240 | .648 | .137 | 1 | .712 | 1.271 | .357 | 4.525 |
| Education_c × SII_c | -.325 | .153 | 4.525 | 1 | **.033** | .722 | .536 | .975 |
| Depression (yes = 1) | .721 | .439 | 2.697 | 1 | .101 | 2.057 | .870 | 4.866 |
| Constant | -5.738 | 1.558 | 13.572 | 1 | <.001 | .003 |  |  |
|  | | | | | | | | |

Note. Theory-driven hierarchical forced-entry binary logistic regression. Predictors entered in five pre-specified blocks: (1) age, sex; (2) clinical covariates (disease duration in months, ALS Functional Rating Scale-Revised [ALSFRS-R] total score at time of testing, site of onset); (3) Education_c, occupation level; (4) Social Interaction Index, marital status, Education_c × SII_c; (5) depression history. Bold red p-values indicate p < .05. Exp(B) = odds ratio; CI = confidence interval. Ste of onset coded 1 = bulbar;. Sex coded 1 = female. Depression coded 1 = history of affective disorder (yes). Marital status coded 1 = partnered. Education_c × SII_c = interaction between mean-centred Education_c and mean-centred SII_c. No premorbid assessment available for ECAS-CI models. Bold red p-values indicate p < .05. Exp(B) = odds ratio; CI = confidence interval. No premorbid assessment available for ECAS-CI models. ECAS-CI Loss of Empathy impairment criterion: subscale meeting clinical threshold.

**Table S6.** *Final model: ECAS-CI Perseverative/Compulsive Behaviour subscale — binary logistic regression*

| **Variable** | **B** | **S.E.** | **Wald** | **df** | **Sign.** | **Exp(B)** | **95% CI Exp(B)** | |
| --- | --- | --- | --- | --- | --- | --- | --- | --- |
|  |  |  |  |  |  |  | **Lower** | **Upper** |
| Sex (female = 1) | .210 | .177 | 1.410 | 1 | .235 | 0.810 | .573 | 1.147 |
| Age | .018 | .008 | 4.933 | 1 | **.026** | 1.019 | 1.002 | 1.035 |
| Education_c (years) | -.024 | .025 | .893 | 1 | .345 | .977 | .930 | 1.026 |
| Occupation level | .059 | .128 | .212 | 1 | .645 | 1.060 | .826 | 1.362 |
| ALSFRS-R | .152 | .227 | .449 | 1 | .503 | 1.164 | .746 | 1.816 |
| Time from onset to testing (months) | .004 | .007 | .298 | 1 | .585 | 1.004 | .990 | 1.018 |
| Site of onset (bulbar = 1) | .379 | .292 | 1.687 | 1 | .194 | 1.460 | .831 | 2.584 |
| SII_c | -.792 | .570 | 1.935 | 1 | .164 | .453 | .148 | 1.383 |
| Marital status (single = 1) | -.456 | .523 | .760 | 1 | .383 | .634 | .227 | 1.767 |
| Education_c × SII_c | .063 | .114 | .309 | 1 | .578 | 1.065 | .852 | 1.331 |
| Depression (yes = 1) | .247 | .371 | .442 | 1 | .506 | 1.280 | .618 | 2.650 |
| Constant | -.848 | 1.053 | .649 | 1 | .421 | .428 |  |  |
|  | | | | | | | | |

Note. Theory-driven hierarchical forced-entry binary logistic regression. Predictors entered in five pre-specified blocks: (1) age, sex; (2) clinical covariates (disease duration in months, ALS Functional Rating Scale-Revised [ALSFRS-R] total score at time of testing, site of onset); (3) Education_c, occupation level; (4) Social Interaction Index, marital status, Education_c × SII_c; (5) depression history. Bold red p-values indicate p < .05. Exp(B) = odds ratio; CI = confidence interval. Ste of onset coded 1 = bulbar;. Sex coded 1 = female. Depression coded 1 = history of affective disorder (yes). Marital status coded 1 = partnered. Education_c × SII_c = interaction between mean-centred Education_c and mean-centred SII_c. No premorbid assessment available for ECAS-CI models. ECAS-CI Perseveration impairment criterion: subscale meeting clinical threshold. Overall model chi-square p = .036.

**Table S7.** *Final model: ECAS-CI Hyperorality/Dietary Changes subscale — binary logistic regression*

| **Variable** | **B** | **S.E.** | **Wald** | **df** | **Sign.** | **Exp(B)** | **95% CI Exp(B)** | |
| --- | --- | --- | --- | --- | --- | --- | --- | --- |
|  |  |  |  |  |  |  | **Lower** | **Upper** |
| Sex (female = 1) | .037 | .203 | .033 | 1 | .855 | 0.963 | .648 | 1.493 |
| Age | .030 | .010 | 8.467 | 1 | **.004** | 1.031 | 1.010 | 1.052 |
| Education_c (years) | -.112 | .031 | 12.973 | 1 | **.001** | .894 | .841 | .950 |
| Occupation level | -.141 | .150 | .891 | 1 | .345 | .868 | .647 | 1.164 |
| ALSFRS-R | .329 | .313 | 1,100 | 1 | .294 | 1.389 | .752 | 2.566 |
| Time from onset to testing (months) | -.001 | .008 | .015 | 1 | .901 | .999 | .983 | 1.015 |
| Site of onset (bulbar = 1) | .011 | .200 | .003 | 1 | .957 | 1.001 | .683 | 1.497 |
| SII_c | -1.003 | .648 | 2.392 | 1 | .122 | .367 | .103 | 1.307 |
| Marital status (single = 1) | .514 | .521 | .973 | 1 | .324 | 1.672 | .602 | 4.645 |
| Education_c × SII_c | -.204 | .136 | 2.238 | 1 | .135 | .816 | .624 | 1.065 |
| Depression (yes = 1) | .231 | .401 | .333 | 1 | .564 | 1.260 | .575 | 2.762 |
| Constant | -2.237 | 1.223 | 3.345 | 1 | .067 | .107 |  |  |
|  | | | | | | | | |

Note. Theory-driven hierarchical forced-entry binary logistic regression. Predictors entered in five pre-specified blocks: (1) age, sex; (2) clinical covariates (disease duration in months, ALS Functional Rating Scale-Revised [ALSFRS-R] total score at time of testing, site of onset); (3) Education_c, occupation level; (4) Social Interaction Index, marital status, Education_c × SII_c; (5) depression history. Bold red p-values indicate p < .05. Exp(B) = odds ratio; CI = confidence interval. Ste of onset coded 1 = bulbar;. Sex coded 1 = female. Depression coded 1 = history of affective disorder (yes). Marital status coded 1 = partnered. Education_c × SII_c = interaction between mean-centred Education_c and mean-centred SII_c. No premorbid assessment available for ECAS-CI models. ECAS-CI Hyperorality/Dietary Changes impairment criterion: subscale meeting clinical threshold.

**Table S8.** *Model fit and discriminative accuracy indices for all ten hierarchical logistic regression models*.

| **Model** | **Instrument** | **Outcome domain** | **n** | **Nagelkerke R²** | **AUC** |
| --- | --- | --- | --- | --- | --- |
| 1 | FrSBe | Total score | 965 | .037 | .685 |
| 2 | FrSBe | Apathy | 965 | .092 | .670 |
| 3 | FrSBe | Disinhibition | 965 | .025 | .743 |
| 4 | FrSBe | Executive dysfunction | 965 | .169 | .751 |
| 5 | ECAS-CI | Total score | 633 | .030 | .585 |
| 6 | ECAS-CI | Apathy/Inertia | 633 | .035 | .557 |
| 7 | ECAS-CI | Disinhibition | 633 | .053† | .642† |
| 8 | ECAS-CI | Loss of Empathy | 633 | .043 | .632 |
| 9 | ECAS-CI | Perseveration | 633 | .027 | .591 |
| 10 | ECAS-CI | Hyperorality | 633 | .103 | .679 |

Note. Nagelkerke R² and AUC are reported for the final model (all blocks entered). AUC = area under the receiver operating characteristic curve, derived from predicted probabilities saved from each logistic regression model. FrSBe models (1–4) include a premorbid baseline component (FrSBe ‘before’ subscale score); ECAS-CI models (5–10) do not. †Model 7 did not achieve convergence due to complete separation caused by marital status; indices should be interpreted with caution.
