# Supplementary material for: Toward a Behavioral Reserve Model in Amyotrophic Lateral Sclerosis
Source: Ann Neurol. 2026 Jul 30;100(5):934–47. doi: 10.1002/ana.78315 (PMC13688070; doi:10.1002/ana.78315)
Supplement: Supplementary file 2 [file ANA-100-934-s002.docx]

**Supplementary methods**

***List of cognitive and behavioral tests*** ***performed for each patient.*** Executive function was tested with Letter Fluency test (FAS), Category Fluency Test (CAT), Trail Making Test B-A (TMT B-A), Frontal Assessment Battery (FAB), ECAS Executive Function score, and ECAS Verbal Fluency score; Language was assessed with ECAS Language subscore and Boston Naming Test; Verbal Memory with Rey Auditory Verbal Learning Test, Immediate Recall (RAVL-IR), Rey Auditory Verbal Learning Test, delayed Recall (RAVL-DR), Babcock Story Recall Test, Immediate Recall (BSRT-IR), Babcock Story Recall Test, Delayed Recall (BSRT-DR), and ECAS Memory score; Visual Memory with Rey-Osterrieth Complex Figure Test, differed recall (ROCF-DR); Visuoconstructive abilities with Rey-Osterrieth Complex Figure Test, Immediate Recall (ROCF-IR), Clock Drawing Test (Clock), and ECAS Visuospatial Abilities score; Attention/Working memory with Digit Span Forward (FW) and Digit Span Backward (BW); Psychomotor speed with Trail Making Test A (TMT A); Non-Verbal Intelligence with Raven’s Colored Progressive Matrices (CPM47); Cognitive flexibility with Trail Making Test B (TMT B); Theory of Mind (social cognition) with Story-based Empathy Task (SET); Behavior with Frontal Systems Behavior Scale (FrSBe), and ECAS Carer Interview (ECAS-CI). For ECAS we used the validated Italian version (Poletti et al 2018), and for the behavioral component of ECAS the cut-off based on number of symptoms (Poletti et al 2023).

***Occupational attainment***. Occupational attainment was assessed according to the protocol proposed by Borroni et al. (2009). Scores range from 0 to 4: (0) no occupation; (1) unskilled laborer or housewife; (2) skilled laborer, tradesperson, lower-level civil servant, employee, self-employed small business owner, or office/sales worker; (3) mid-level civil servant or manager, head of a small business, academic, or specialist in a subordinate position; (4) senior civil servant or manager, senior academic position, or self-employed individual with a high degree of responsibility. Unlike the original study, we considered each participant’s highest level of employment.
